# Functional microarray analysis of differentially expressed genes in granulosa cells from women with polycystic ovary syndrome related to MAPK/ERK signaling

**DOI:** 10.1038/srep14994

**Published:** 2015-10-13

**Authors:** Chen-Wei Lan, Mei-Jou Chen, Kang-Yu Tai, Danny CW Yu, Yu-Chieh Yang, Pey-Shynan Jan, Yu-Shih Yang, Hsin-Fu Chen, Hong-Nerng Ho

**Affiliations:** 1 Graduate Institute of Clinical Medicine, College of Medicine, National Taiwan University Taipei, Taiwan; 2 Division of Reproductive Endocrinology and Infertility, Department of Obstetrics and Gynecology, College of Medicine and the Hospital, National Taiwan University Taipei, Taiwan; 3 Graduate Institute of Medical Genomics and Proteomics, College of Medicine, National Taiwan university Taipei, Taiwan; 4 Genome and Systems Biology Degree Program, National Taiwan University Taipei, Taiwan

## Abstract

Polycystic ovary syndrome (PCOS) is the most common endocrine disorder in women of reproductive age. Although its aetiology and pathogenesis remain unclear, recent studies suggest that the dysfunction of granulosa cells may partly be responsible. This study aimed to use cDNA microarray technology to compare granulosa cell gene expression profiles in women with and without PCOS to identify genes that may be aetiologically implicated in the pathogenesis of PCOS. The study cohort included 12 women undergoing *in vitro* fertilization, six with PCOS and six without PCOS. Differential gene expression profiles were classified by post-analyses of microarray data, followed by western blot analyses to confirm the microarray data of selected genes. In total, 243 genes were differentially expressed (125 upregulated and 118 downregulated) between the PCOS and non-PCOS granulosa cells. These genes are involved in reproductive system development, amino acid metabolism and cellular development and proliferation. Comparative analysis revealed genes involved in the mitogen-activated protein kinase/extracellular regulated kinase (MAPK/ERK) signaling pathways. Western blot analyses confirmed that mitogen-activated protein kinase kinase kinase 4 and phospho-ERK1/2 were decreased in PCOS granulosa cells. This study identified candidate genes involved in MAPK/ERK signaling pathways that may influence the function of granulosa cells in PCOS.

Polycystic ovary syndrome (PCOS) is a common heterogeneous endocrinopathy characterised by hyperandrogenemia (HA), arrested ovarian follicle development, menstrual irregularity and polycystic ovaries that results in anovulation and infertility in women of reproductive age^1^,^2^,^3^. Furthermore, PCOS is a leading cause of several other health problems in women, including menstrual dysfunction, hirsutism, acne, obesity, and metabolic syndrome^4^,^5^,^6^,^7^,^8^. Although evidence suggests that PCOS has a complex aetiology with a genetic basis, its exact genetic aetiology remains unclear^9^,^10^.

Granulosa cells are important in ovarian folliculogenesis as they provide a suitable microenvironment for follicular development and oocyte maturation. In response to pituitary gonadotropin secretion, granulosa cells upregulate the expression of a variety of genes that encode the components of steroidogenic pathways involved in estrogen biosynthesis^11^. Recent studies have indicated that in women with PCOS, granulosa cell dysfunction may contribute to abnormal folliculogenesis, excess production of intraovarian androgens and/or increased circulating anti-Müllerian hormone levels, although the underlying mechanisms remain to be elucidated^12^,^13^,^14^,^15^,^16^,^17^. Therefore, it is clinically important to explore the mechanisms underlying the dysfunction of granulosa cells in women with PCOS.

DNA microarray technology is a suitable modality to examine changes in global gene expression among granulosa cells in PCOS and may aid in the identification of novel candidate genes that contribute to the aetiology of PCOS. Previous studies comparing gene expression arrays in theca cells, ovarian cells, oocytes, cumulus cells and granulosa cells between women with PCOS and without PCOS have identified several candidate pathways underlying differential gene expression among various cell types^18^,^19^,^20^,^21^,^22^,^23^,^24^. However, to the best of our knowledge, gene expression profiles and identification of mechanisms in granulosa-lutein cells isolated from normal-weight Asian women with PCOS undergoing *in vitro* fertilization (IVF) have not yet been reported^22^.

Therefore, we compared the gene expression profiles of non-PCOS and PCOS granulosa cells from preovulatory follicles using Affymetrix® microarray chips to assess global changes in granulosa cell gene expression profiles and alterations in genetic networks, signal transduction, transcription/translation and/or metabolic pathways critical to PCOS granulosa cell phenotypes that may impact oocyte maturation and follicular development.

## Materials and Methods

### Patient selection and ethical statement

All study participants were women undergoing IVF at the Center for Reproduction at the National Taiwan University Hospital (Taipei City, Taiwan). A diagnosis of PCOS was based on the Rotterdam diagnostic criteria^3^: (I) chronic oligo-ovulation and/or anovulation (cycle lengths >35 days); (II) biochemical HA (serum total testosterone levels ≧0.8 ng/mL) and/or clinical HA, including hirsutism, acne and/or alopecia; and (III) polycystic ovaries on ultrasound. PCOS was defined when at least two of these three features were present. We enrolled 12 women in this study (Table 1), six with PCOS (PCOS group) and six without PCOS (non-PCOS group) with male factor infertility as the control group. Among the six women in the PCOS group, six had chronic oligo-ovulation (6/6), three had biochemical HA (3/6), six had clinical HA (6/6) and all six had polycystic ovaries (6/6). Each of the six women with PCOS satisfied all three Rotterdam criteria. The average patient age was 34 years (range, 31–37 years). A luteinizing hormone/follicle-stimulating hormone (LH/FSH) ratio of >1 was accepted as the supplemental biochemical evidence of PCOS. All six women with PCOS had an LH/FSH ratio of >1. In this study, we recruited only women with a normal body mass index (BMI) of 19–24 kg/m^2^ to minimize the effects of obesity and fasting blood glucose (glucose AC, 70–100 mg/dL). The study protocol was approved by the Research Ethics Committee of the National Taiwan University Hospital (approval no.: NTUH-REC No.: 201212125RIND) and conducted in accordance with approved institutional guidelines. All participants provided signed informed consent.

### Assays

After overnight fasting, venous blood samples were collected in the early follicular phase from all subjects, allowed to clot for 30 min and then centrifuged at 2500 rpm for approximately 10 min to obtain serum. Serum total testosterone levels [detectable range (DR): 0.03–14.4 ng/mL] were measured using radioimmunoassay (Diagnostic Systems Laboratories, Webster, TX, USA)^25^. The concentration of fasting blood glucose (glucose AC, DR: 10–800 mg/dL), FSH (DR: 0.1–120 mIU/mL), LH (DR: 0.1–100 mIU/mL) and oestradiol (E2, DR: 20–900 pg/mL) were measured as described previously^25^,^26^,^27^.

### Ovarian stimulation, granulosa cell collection and RNA extraction

During ovarian stimulation, the dose of gonadotropin (Merck Serono S.A., Geneva, Switzerland) was adjusted for each individual patient according to FSH levels, serum E2 concentrations and growth and number of follicles. Follicular fluid was aspirated and pooled from small antral follicles (4–8 mm in diameter) visible on ultrasonography during the retrieval of transvaginal oocytes for IVF. The granulosa cells were isolated and prepared from follicular aspirates after the removal of oocytes. The samples were then centrifuged at 400 × g for 10 min to separate the fluid from cells. The cell pellets were resuspended in Dulbecco’s phosphate-buffered saline (DPBS; GIBCO®; Invitrogen Life Technologies GibcoWaltham, MA, USA), layered on 50% Ficoll–Paque™ density gradient media (GE Healthcare, Waukesha, WI, USA) and then centrifuged at 400 × for 30 min to remove maximum red blood cells. The granulosa cells at the interface were collected and washed with DPBS. Total RNA was extracted from individual patient samples using the RNeasy® Mini Kit (Qiagen, Inc., Valencia, CA, USA) according to the manufacturer’s instructions and then treated with deoxyribonuclease. The concentration of isolated RNA was determined using a NanoDrop® ND-100 spectrophotometer (NanoDrop Technologies, LLC, Wilmington, DE, USA). The RNA extracts were stored at −80 °C until all samples were collected. The quality and integrity of RNA samples were analyzed using the Agilent 2100 Bioanalyzer (Agilent Technologies, Inc., Palo Alto, CA, USA).

### Microarray hybridization analysis

Affymetrix® Gene Chip Human Genome Gene 1.0 ST microarray chips (Affymetrix®, Inc., Santa Clara, CA, USA) were hybridized at the National Health Research Institutes Microarray Core Facility (Miaoli County, Taiwan). Briefly, total RNA was reverse transcribed into double-stranded cDNA using an oligo^24^ primer containing a T7 RNA polymerase promoter. Single-stranded cDNA labeled with biotinylated nucleotides in the sense direction were generated from cRNA and used for array hybridization. The arrays were washed and stained using the Affymetrix® Fluidics Station 450 and then scanned using the Affymetrix® GeneArray 3000 7 G scanner to generate fluorescent images. Cell intensity data (CEL files) were generated using Affymetrix® GeneChip® Command Console® software.

### Microarray data annotation and ingenuity pathway analysis (IPA)

Data were normalized using quantile normalization methods^28^. A list of differentially expressed genes was generated by Linear Models for Microarray (limma^29^ packages in the R platform) data analysis, and their corrected P values were calculated using the Benjamini–Hochberg method^30^ (i.e. false discovery rate) for the non-PCOS group compared with the PCOS group and uploaded to the IPA Ingenuity System (http://www.ingenuity.com) to identify gene networks. Gene networks were used to identify biologic functions, molecular processes, disorders and pathways of genes identified by Limma data analysis.

### Protein extraction and western blot analyses

Total protein was extracted from an additional three non-PCOS granulosa cells and three PCOS granulosa cells, and 20 μg of protein per lane was loaded and separated by sodium dodecyl sulfate-polyacrylamide gel electrophoresis. Proteins were then transferred to polyvinylidene fluoride (PVDF) membranes and blocked for 1 h with 5% nonfat milk in Tris-buffered saline with Tween-20 (Gibco). The membranes were incubated overnight at 4 °C with one of the following primary antibodies: phosphorylated extracellular signal-regulated kinase (p-ERK; diluted 1:500), ERK (diluted 1:1000), mitogen-activated protein kinase kinase kinase 4 (MAP3K4; diluted 1:500), or glyceraldehyde 3-phosphate dehydrogenase (GAPDH; diluted 1:5000) (Santa Cruz Biotechnology, Inc., Santa Cruz, CA, USA). Next, PVDF membranes were incubated for 60 min at room temperature with secondary antibodies (Santa Cruz Biotechnology, Inc.) and then immune complexes were detected by chemiluminescence using a western blotting analysis system (NENTM Life Science, Boston, MA, USA). Relative protein levels per sample were then normalized to GAPDH signals.

### Statistical analysis

Clinical features of interest were selected as dependent variables. The Mann–Whitney *U* test, Student’s *t*-test and paired *t*-test of variance were used where appropriate for statistical analyses. Data from at least three independent measurements are presented as means ± standard deviation of the mean (SEM). The SYSTAT statistical graphics software package (SYSTAT Software, Inc., San Jose, CA, USA) was used for analysis, and a probability (P) value of <0.05 was considered statistically significant.

## Results

### The clinical and hormonal characteristics of patients with and without PCOS

The granulosa cells were isolated from six women without PCOS and six women with PCOS undergoing IVF. The clinical and biochemical characteristics of study participants are listed in Table 1. There were no differences between the groups in regard to age; mean BMI; basal FSH, LH and E2 levels; glucose AC; duration of ovarian stimulation or total FSH dose. The mean menstrual cycle length, LH/FSH ratio, number of aspirated follicles and total testosterone levels were significantly higher in the PCOS group than in the non-PCOS group.

### Profiles of differentially expressed genes

**Figure 1:**
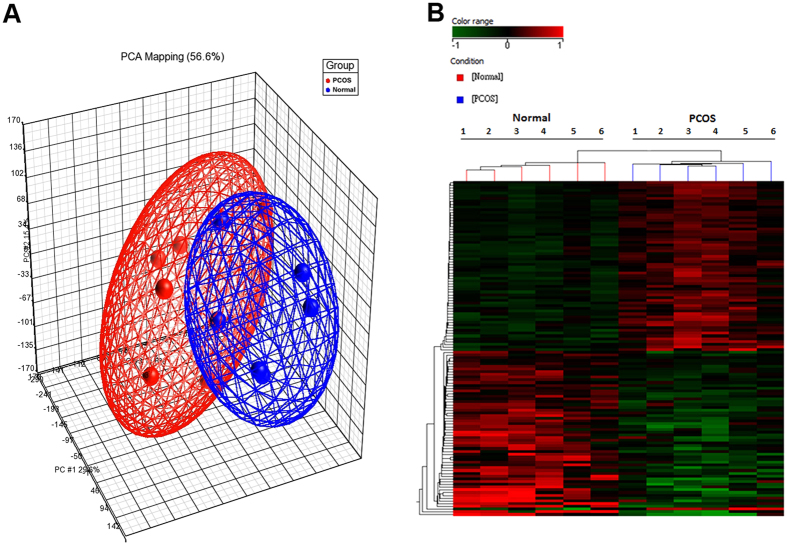
Poly component analysis and hierarchical cluster of differentially expressed genes in granulosa cells of the non-PCOS controls (n = 6) vs. patients with PCOS (n = 6). (**A**) Principal component analysis (PCA) of entire gene expression levels of all samples. (**B**) Hierarchical cluster analysis performed on the profiles of 243 differentially expressed genes.

All 12 samples were analyzed to select genes that were significantly upregulated or downregulated. A total of 243 genes were differentially expressed in the granulosa cells from the non-PCOS group compared with those from the PCOS group (Supplemental information 1). Differentially expressed genes were visualized by hierarchical clustering and principal component analysis (Fig. 1A,B). Compared with the non-PCOS group, 118 genes were significantly downregulated and 125 were significantly upregulated in the PCOS group. The 10 most upregulated and downregulated genes in the non-PCOS group compared with the PCOS group are listed in Table 2.

### Function, network and signal transduction pathways with significantly altered gene expression profiles in granulosa cells from women with PCOS

**Figure 2:**
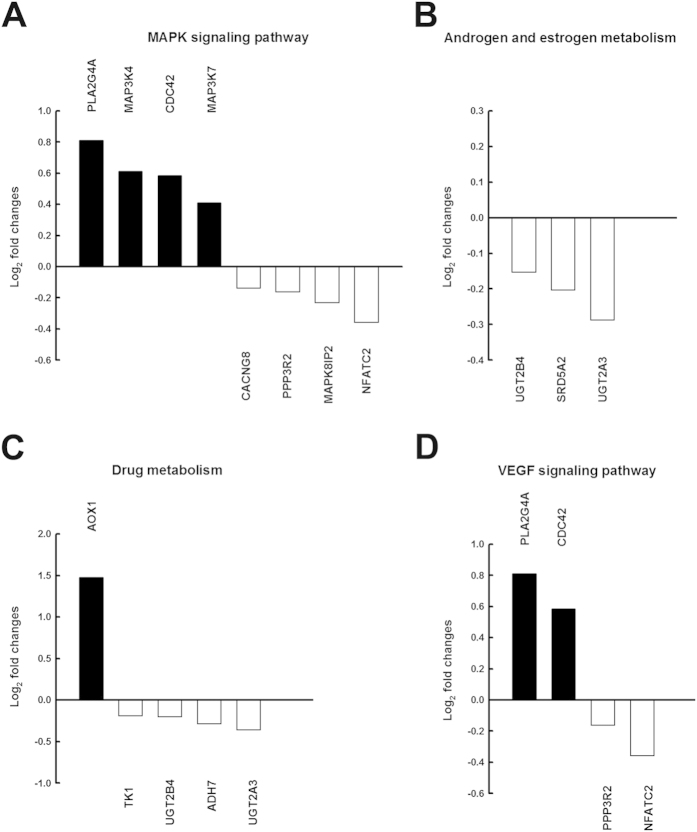
Functional and mechanism analysis of microarray data indicated differential expression according to the Kyoto encyclopedia of genes and genomes (KEGG) for the non-PCOS controls (n = 6) vs. patients PCOS (n = 6). (**A**) Mitogen-activated protein kinases (MAPK) signaling pathway, (**B**) androgen and estrogen metabolism, (**C**) drug metabolism, and (**D**) vascular endothelial growth factor (VEGF) signaling pathway in the granulosa cells. Black bars represent genes with greater expression in the non-PCOS controls; white bars represent genes with greater expression in PCOS.

**Figure 3:**
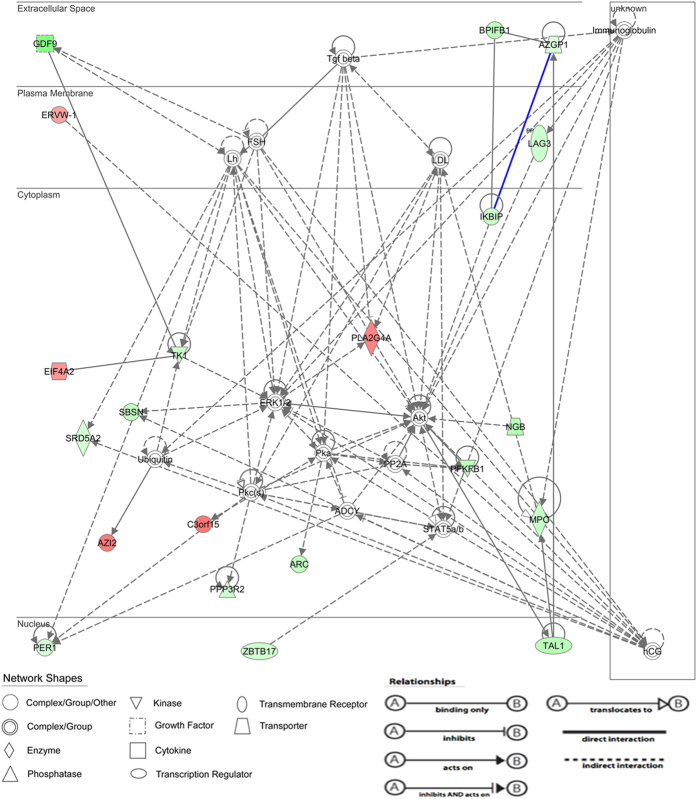
Reproduction system network of genes that were either upregulated or downregulated in the non-PCOS compared with PCOS granulosa cells. Genes are arranged into 4 horizontal compartments (nucleus, cytoplasm, plasma membrane, and extracellular space). The differences in color intensity of the molecules show the higher or lower expression.

The functional differences between the two groups were used to construct a differential gene–protein interaction network of 243 differentially expressed genes identified in granulosa cells. Kyoto encyclopedia of genes and genomes (KEGG) pathway analysis suggested that these genes were typically involved in MAPK signaling, VEGF signaling, androgen and estrogen metabolism and drug metabolism (Fig. 2). Of a total of eight MAPK signaling pathway genes that were identified in the non-PCOS granulosa cells, four were comparatively upregulated and four downregulated compared with the PCOS granulosa cells (Fig. 2A). In the PCOS group, we also identified three androgen and estrogen metabolism-related genes that were downregulated (Fig. 2B), five drug metabolism-related genes (one upregulated and four downregulated; Fig. 2C) and four VEGF signaling pathway genes (two upregulated and two downregulated; Fig. 2D). In addition, IPA analysis revealed involvement in the following cellular functions: reproductive system development and function; amino acid metabolism; cell morphology; hereditary disorder; cell-to-cell signaling and interaction; cellular development and proliferation; cell cycle and DNA replication, recombination and repair. The top network represented in differentially expressed genes was the reproductive system development and function and amino acid metabolism network (score = 38) in the non-PCOS granulosa cells compared with the PCOS granulosa cells. The reproductive system development and function network demonstrated high representation of the following genes (Fig. 3): sexual reproduction genes, phospholipase A2, group IVA (cytosolic, calcium-dependent, PLA2G4A), steroid-5-alpha-reductase, alpha polypeptide 2 (3-oxo-5 alpha-steroid delta 4-dehydrogenase alpha 2, SRD5A2), thymidine kinase 1, soluble TK1 and growth differentiation factor 9 (GDF9) as well as reproduction genes involved in the MAPK/ERK pathway. In addition, this reproductive system development and function network was related by steroidogenic genes, LH, FSH, human chorionic gonadotropin (hCG) and low-density lipoprotein (Fig. 3).

### Validation of microarray data by western blot analysis: MAP3K4 expression and ERK1/2 phosphorylation was reduced in the PCOS granulosa cells

**Figure 4:**
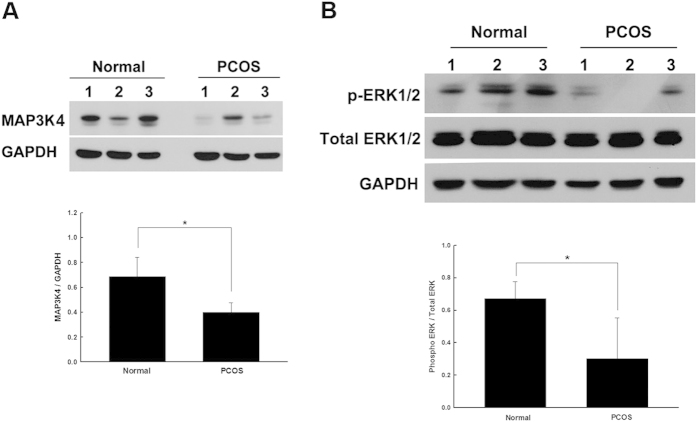
The activation of MAP3K4 and p-ERK1/2 was decreased in PCOS granulosa cells when compared with the non-PCOS granulosa cells. Western blot analysis of the MAP3K4 protein (**A**) and the p-ERK1/2 protein (**B**) in the non-PCOS granulosa cells and PCOS granulosa cells. Glyceraldehyde 3-phosphate dehydrogenase (GAPDH), internal control for quantitation. Bar chart represents the quantitation of protein levels. *P < 0.05. Bars represent means ± standard deviations (SD) of data from ≥3 independent experiments. In (**A**,**B**) relevant parts of Western blot images were cropped from full-length blots shown in Fig. S1.

In this study, we coupled the KEGG and IPA data to identify statistically significant differences in MAPK/ERK signaling between the non-PCOS and PCOS granulosa cells. To further validate whether there were differences at the protein level, we compared MAP3K4 expression in granulosa cells obtained from three other independent patients from each group by western blot analysis and observed a 30% reduction in MAP3K4 expression in the PCOS granulosa cells (Fig. 4A). The most widely studied MAPK pathway is the ERK cascade, of which ERK1 and ERK2 form the central components and have been demonstrated to be associated with steroidogenesis in granulosa cells^31^,^32^. Furthermore, we examined the downstream molecular mechanisms of both p-ERK1/2 and ERK1/2 by western blot analysis in three independent patients each from the non-PCOS and PCOS groups. Our analysis showed that p-ERK1/2 was comparatively decreased in the PCOS granulosa cells (Fig. 4B). A comparison of MAP3K4 and p-ERK1/2 revealed that both were reduced in the PCOS granulosa cells compared with the non-PCOS granulosa cells. These results concur with the data obtained by the microarray assay at the protein level.

## Discussion

The purpose of this study was to use DNA microarray data to identify differentially expressed genes in the granulosa cells of women with and without PCOS to provide novel information for the further study of associated disease mechanisms and also to bring about advancement in disease treatment. These microarray profiles indicated that the levels of activated MAP3K4 and p-ERK1/2 were reduced in the PCOS granulosa cells, which are potentially correlated with reproduction. These findings provide further evidence that the MAPK signaling pathway plays an important role in the pathogenesis of PCOS. Furthermore, these differentially expressed genes were also involved in androgen and estrogen metabolism, drug metabolism and VEGF signaling.

A limitation to this genetic study of PCOS was the relatively small number of subjects, which was due to the limited clinical access to patient tissues. PCOS is a genetically complex heterogeneous endocrinopathy affecting 5%–10% of reproductive age women. The strength of this study was that PCOS was assessed only among women of reproductive age (average age, 34 years) who were undergoing IVF. It was particularly difficult to collect sufficiently large cohorts of such patients because our recruitment of women with PCOS was based on the Rotterdam diagnostic criteria and only those with a normal BMI of 19–24 kg/m^2^ and glucose AC level of 70–100 mg/dL were included to minimize the effects of obesity and insulin resistance.

We used microarray analysis, IPA and KEGG data to identify several important pathways, biological function networks and groups of significant candidate PCOS genes. We also compared pathways to identify common, unique and unions of pathway participants by calculating and comparing the significance of various potential genes between our cases and controls. Although differences in gene expression profiles were fairly small, the significance fulfils the criteria of the IPA software for further comprehensive research of these unique genes.

Among the 10 most highly differentially expressed genes detected in this study, some were previously shown to be associated with PCOS, including aldehyde oxidase 1, DEAH (Asp-Glu-Ala-His) box polypeptide 16, chromosome 1 open reading frame 54 and F-box protein 5^19^,^21^,^33^,^34^. The 7SK small nuclear RNA is reportedly expressed in male testes and is also highly expressed in female ovaries^35^. In addition, we observed that B-cell lymphoma 2-like 15 (BCL2L15), which reportedly plays important roles in folliculogenesis and oocyte maturation^36^, was among the top upregulated genes. Notably, the BCL2 family proteins are the key regulators of apoptotic processes that govern human oocyte development and survival^36^,^37^. Therefore, we propose that the observed low levels of BCL2L15 in the PCOS granulosa cells compared with those in the non-PCOS granulosa cells may be involved in an increased risk of PCOS.

Our results also indicated that a group of genes that were upregulated or downregulated in the PCOS granulosa cells were involved in the reproductive system development and function. These genes included the transcription regulators, T-cell acute lymphocytic leukemia 1 and zinc finger and BTB domain containing 17; the transporters, neuroglobin and alpha-2-glycoprotein 1and the zinc-binding proteins, PLA2G4A, SRD5A2, TK1, GDF9, myeloperoxidase, transmembrane receptor lymphocyte-activation gene 3 and 6-phosphofructo-2-kinase/fructose-2,6-biphosphatase 1. The PLA2G4A enzyme is stimulated by the preovulatory LH/hCG surge in granulosa cells of ovulatory follicles^38^. SRD5A2 (enzyme 5α-reductase) plays an important role in the steroid metabolism cascade to convert testosterone and cortisol to dihydrotestosterone and dihydrocortisol, respectively^39^. Previous research has indicated that decreased SRD5A2 activity is associated with the pathogenesis of PCOS, even in women with normal weight. Therefore, an increase in the activity of this enzyme might decrease cortisol production with a consequent increase in androgen levels. Together, these observations suggest that SRD5A2 may influence ovarian function and promote the symptoms of HA in PCOS, such as hirsutism, acne and alopecia^40^,^41^. The abnormal expression of the enzyme TK is significantly associated with the incidence of PCOS^42^. GDF9 is an oocyte-specific growth factor that plays key roles in oocyte–granulosa cell communication during folliculogenesis^43^, and its dysregulation may contribute to aberrant folliculogenesis in PCOS^44^. In addition, these genes regulate reproductive development and function via several signaling pathways, including MAPK/ERK, Akt, signal transducer and activator of transcription 5 and transforming growth factor beta. The MAPK/ERK pathway consists of a chain of cell proteins that facilitate signal transduction from the cytosol to the nucleus for the regulation of cell survival, proliferation and differentiation through phosphorylation cascades^45^,^46^. Previous studies have demonstrated that alternative MAPK/ERK signaling pathways are associated with FSH-induced steroid biosynthesis in granulosa cells^47^,^48^. In addition, other studies have determined that the abnormal activation of the MAPK/ERK signaling pathway in different tissues may contribute to defects observed in metabolic signaling and excessive ovarian androgen production in women with PCOS^49^,^50^. Our microarray data showed that MAP3K4 was downregulated in the PCOS granulosa cells compared with the non-PCOS granulosa cells. IPA analysis revealed that the top reproductive system development and function network (score = 38) was correlated with the MAPK/ERK signaling pathway. On the basis of these results, we further investigated MAP3K4 signaling and determined that downstream p-ERK1/2 expression was decreased in the PCOS granulosa cells at the protein level. MAP3K4 is an MAPK that activates the p38 MAPK and c-Jun N-terminal kinase signaling pathways in embryonic gonadal somatic cells and regulates embryonic gonadal activity^51^,^52^. This study is the first to indicate an association of MAP3K4 activation with PCOS and to show that it is involved in the MAPK/ERK signaling pathway. Importantly, the microarray gene expression profiles identified in this study give further insight into the molecular mechanisms underlying the pathogenesis of PCOS. However, future studies will be required to clarify the interaction of the MAPK/ERK signaling pathways in the granulosa cell pathophysiology in PCOS.

In conclusion, we identified several genes that were differentially expressed between non-PCOS and PCOS granulosa cells. The present data show for the first time that expression of the novel player MAP3K4 was decreased in the PCOS granulosa cells and participated in the MAPK/ERK signal transduction pathway. These differentially expressed genes are involved in various biological functions, particularly reproduction and development, and provide a mechanism through which granulosa cells may contribute to the pathogenesis of PCOS. These results will help to further clarify the molecular basis and the functional significance of several novel genes involved in the pathogenesis of PCOS.

## Additional Information

**How to cite this article**: Lan, C.-W. *et al.* Functional microarray analysis of differentially expressed genes in granulosa cells from women with polycystic ovary syndrome related to MAPK/ERK signaling. *Sci. Rep.* **5**, 14994; doi: 10.1038/srep14994 (2015).

## Supplementary Material

Supplementary Information

Supplementary Dataset 1

## Figures and Tables

**Table 1:** Clinical parameters in normoovulatory controls and patients with PCOS.

	Normal (n = 6)	PCOS (n = 6)	*P*value
	Mean (±S.D.)	Mean (±S.D.)	
Age (yr)	34.8 (±2.9)	34.3 (±3.3)	0.712
BMI (kg/m^2^)	20.6 (±1.8)	20.0 (±1.0)	0.675
Menstrual cycle length	28.3 (±0.8)	43.2 (±13.0)	0.002**
E2 (pg/mL)	40.7 (±20.7)	32.2 (±18.7)	0.305
LH (mIU/mL)	4.5 (±3.5)	6.2 (±2.6)	0.368
FSH (mIU/mL)	7.9 (±4.9)	5.0 (±1.9)	0.305
LH/FSH (mIU/mL)	0.5 (±0.1)	1.3 (±0.5)	0.009**
Number of aspired follicles	9.5 (±0.5)	31.2 (±3.9)	0.002**
Total testosterone (ng/mL)	0.3 (±0.1)	1.0 (±0.6)	0.015*
Glucose AC (mg/dL)	84.7 (±4.3)	82.8 (±9.0)	0.240
Total FSH dose (IU)	1800.0 (±158.1)	1579.2 (±386.1)	0.355

Significance was tested using Mann-Whitney *U* test. *P < 0.05, **P < 0.01, Normal vs. PCOS subjects. BMI, Body mass index. E2, Estradiol.

**Table 2:** The top ten upregulated and downregulated genes in granulosa cells of normal group compared with PCOS group.

Genes Symbol	Genes Name	log fold change	adj *P* value
Upregulated			
AOX1	aldehyde oxidase 1	1.475	0.001813**
C1orf54	chromosome 1 open reading frame 54	1.311	0.002377**
SNORD59A	small nucleolar RNA, C/D box 59A	1.257	0.002768**
TRIM22	tripartite motif-containing 22	1.212	0.002481**
RN7SK	RNA, 7SK small nuclear	1.180	0.001813**
ANKRD36B	ankyrin repeat domain 36B	1.157	0.001583**
ENPP5	ectonucleotide pyrophosphatase/phosphodiesterase 5	1.115	0.002160**
SLC25A30	solute carrier family 25, member 30	1.085	0.002398**
SNORD21	small nucleolar RNA, C/D box21	1.028	0.001583**
ALPK1	alpha-kinase 1	1.008	0.001583**
Downregulated			
DHX16	DEAH box polypeptide 16	−0.762	0.004345**
FBXO5	F-box protein 5	−0.709	0.013218*
OR14C36	olfactory receptor, family 14, subfamily C,member 36	−0.627	0.007862**
PCOTH	prostate collagen triple helix	−0.613	0.001583**
RPL4	ribosomal protein L4	−0.563	0.003660**
BCL2L15	BCL2-like15	−0.552	0.002969**
OR52E8	olfactory receptor, family 52, subfamily E, member 8	−0.453	0.014957*
GTF2H1	general transcription factor IIH, polypeptide 1	−0.430	0.025261*
OR5A2	olfactory receptor, family 5, subfamily A, member 2	−0.429	0.011000*
PTH2R	parathyroid hormone 2 receptor	−0.403	0.011824*

The adjusted *P* values were shown by False Discovery Rate (FDR) calculation based on the *Benjamini-Hochberg* method^30^. *P < 0.05, **P < 0.01.
