# Supplementary material for: Functional microarray analysis of differentially expressed genes in granulosa cells from women with polycystic ovary syndrome related to MAPK/ERK signaling
Source: Sci Rep. 2015 Oct 13;5:14994. doi: 10.1038/srep14994 (PMC4602237; doi:10.1038/srep14994)

Title: Functional microarray analysis of differentially expressed genes in granulosa cells from women with polycystic ovary syndrome related to MAPK/ERK signaling

Author: Chen-Wei Lan[1,2], Mei-Jou Chen[2], Kang-Yu Tai[4], Danny CW Yu[2], Yu-Chieh Yang[2], Pey-Shynan Jan[2] , Yu-Shih Yang [2], Hsin-Fu Chen[1,2,3*], Hong-Nerng Ho[1,2,3*]

Supplementary Figure S1 Full-length blots for the data in Figure 4.

Fig. 4 (A)

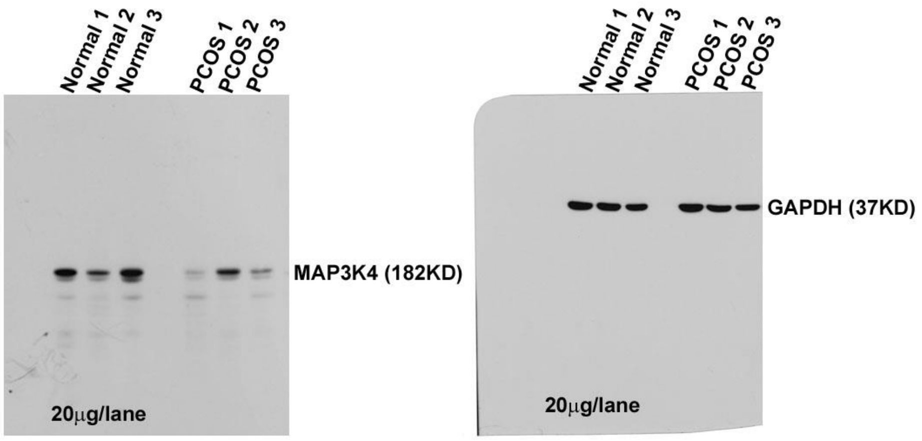

Fig. 4 (B)

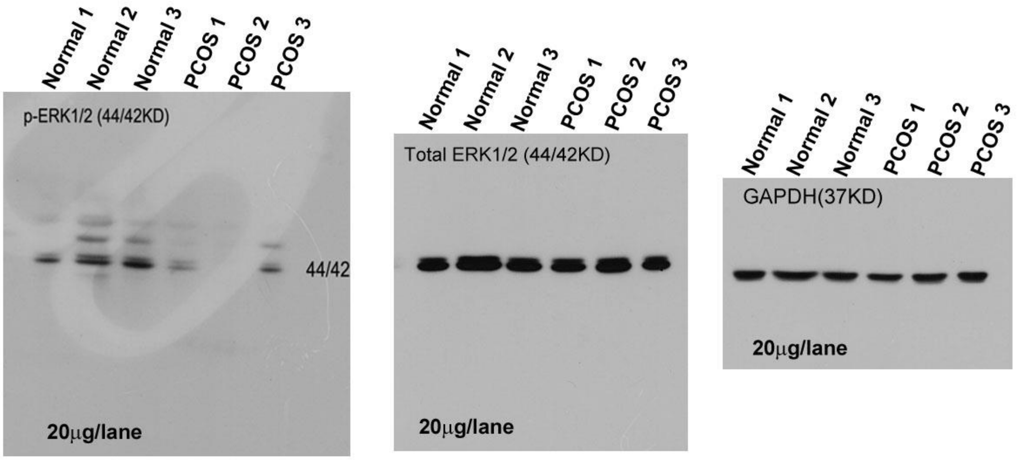

Supplement: Supplementary Information [file srep14994-s1.pdf]
